# Design of new anti-Alzheimer drugs: ring-expansion synthesis and synchrotron X-ray diffraction study of dimethyl 4-ethyl-11-fluoro-1,4,5,6,7,8-hexa­hydro­azonino[5,6-*b*]indole-2,3-di­carboxyl­ate

**DOI:** 10.1107/S2056989018001329

**Published:** 2018-02-07

**Authors:** Flavien A. A. Toze, Anna V. Listratova, Leonid G. Voskressensky, Natalia Yu. Chernikova, Nikolai N. Lobanov, Alexey N. Bilyachenko, Pavel V. Dorovatovskii

**Affiliations:** a Department of Chemistry, Faculty of Sciences, University of Douala, PO Box 24157, Douala, Republic of , Cameroon; b Organic Chemistry Department, Peoples’ Friendship University of Russia, 6 Miklukho-Maklaya St., Moscow 117198, Russian Federation; c Chemistry and Biology Department, Peoples’ Friendship University of Russia, 6 Miklukho-Maklay St., Moscow 117198, Russian Federation; d Inorganic Chemistry Department, Peoples’ Friendship University of Russia, 6 Miklukho-Maklay St., Moscow 117198, Russian Federation; e National Research Centre "Kurchatov Institute", 1 Acad. Kurchatov Sq., Moscow 123182, Russian Federation

**Keywords:** crystal structure, natural alkaloids, azonino­indoles, Alzheimer disease, synchrotron radiation

## Abstract

The title compound, C_20_H_23_FN_2_O_4_, is the product of a ring-expansion reaction from a seven-membered fluorinated hexa­hydro­azepine to a nine-membered azonine. The nine-membered azonine ring of the mol­ecule adopts a *chair–boat* conformation. The C=C and C—N bond lengths [1.366 (3) and 1.407 (3) Å, respectively] indicate the presence of conjugation within the enamine CH_2_—C=C—N—CH_2_ fragment. The substituent planes at the C=C double bond of this fragment are twisted by 16.0 (3)° as a result of steric effects. The amine N(Et) N atom has a trigonal–pyramidal configuration (sum of the bond angles = 346.3°). The inter­planar angle between the two carboxyl­ate substituents is 60.39 (8)°. In the crystal, mol­ecules form zigzag chains along [010] by inter­molecular N—H⋯O hydrogen-bonding inter­actions, which are further packed in stacks toward [100]. The title azonino­indole might be considered as a candidate for the design of new Alzheimer drugs.

## Abstract 2

The nine-membered azonino­indole dimethyl 4-ethyl-11-fluoro-1,4,5,6,7,8-hexa­hydro­azonino[5,6-*b*]indole-2,3-di­carboxyl­ate, representing a candidate for the design of new Alzheimer drugs, has been studied by synchrotron X-ray diffraction.

## Chemical context   

Eight-, nine-, and ten-membered heterocycles, often referred to as medium-sized rings, remain largely unexplored because of the lack of general convenient routes for their synthesis. Meanwhile, such medium-sized heterocycles, in particular azonine, frequently occur in natural products, such as alkaloids (Neuss *et al.*, 1959 ▸, 1962 ▸; Uprety & Bhakuni, 1975 ▸), and thus they are considered to be promising fragments in drug design.

Voskressensky and his group have pioneered the tandem transformation of fused tetra­hydro­pyridines into azines bearing an enamine moiety in the eight-membered ring under the action of activated alkynes. Based on this reaction, convenient preparative routes to tetra­hydro­pyrrolo­[2,3-*d*]azocines (Varlamov *et al.*, 2002 ▸), tetra­hydro­azocino[5,4-*b*]indoles, and tetra­hydro­azocino[4,5-*b*]indoles (Voskressensky *et al.*, 2004 ▸) have been elaborated. The application of a similar approach to hexa­hydro­azepine gives rise to azonino­indoles (Nguyen *et al.*, 2017 ▸), which are otherwise hard to obtain.

**Figure 1:**
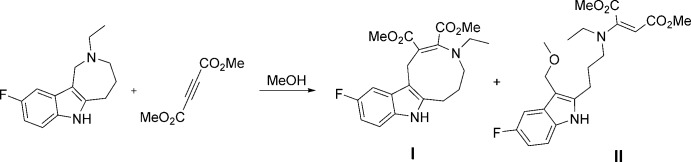
The synthesis of dimethyl 4-ethyl-11-fluoro-1,4,5,6,7,8-hexa­hydro­azonino[5,6-*b*]indole-2,3-dicarboxyl­ate **I** in methanol.

Azonino­indole **I** was successfully synthesized from the initial 2-ethyl-9-fluoro-1,2,3,4,5,6-hexa­hydro­azepino[4,3-*b*]indole *via* a domino reaction under the action of dimethyl acetyl­enedi­carboxyl­ate in methanol at room temperature (Fig. 1 ▸). The domino reaction results in the expansion of the hexa­hydro­azepine ring to the azonine *viz.* dimethyl 4-ethyl-11-fluoro-1,4,5,6,7,8-hexa­hydro­azonino[5,6-*b*]indole-2,3-dicarb­oxyl­ate (**I**). 3-Meth­oxy­methyl-substituted indole **II** was isolated as a by-product of this reaction.

The azonine systems, as a result of their specific structure, are known to act as ligands towards different receptors, thus demonstrating diverse types of biological activity (Magnus *et al.*, 1987 ▸; Kuehne, Bornman *et al.*, 2003 ▸; Kuehne, He *et al.*, 2003 ▸; Afsah *et al.*, 2009 ▸; Rostom, 2010 ▸; Tanaka *et al.*, 2014 ▸; Soldi *et al.*, 2015 ▸; Hartman & Kuduk, 2016 ▸), including anti-Alzheimer’s disease activity (Nguyen *et al.*, 2017 ▸).

**Figure 2:**
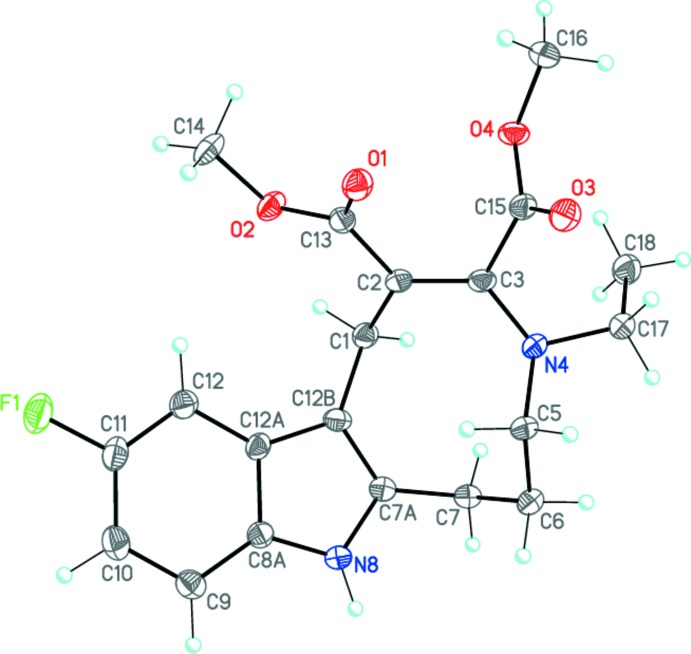
The mol­ecular structure of **I**. Displacement ellipsoids are drawn at the 50% probability level. H atoms are shown as small spheres of arbitrary radius.

The title compound **I**, C_20_H_23_FN_2_O_4_, is the product of a ring expansion reaction from a seven-membered fluorinated hexa­hydro­azepine to a nine-membered azonine. The mol­ecular structure of **I** is unambiguously confirmed by the X-ray diffraction study (Fig. 2 ▸).
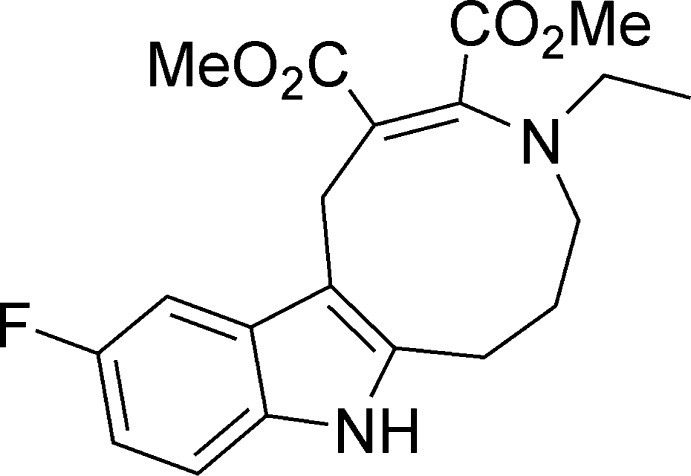


## Structural commentary   

Compound **I** is isostructural to the non-fluorinated analog published by us very recently (Nguyen *et al.*, 2017 ▸). The nine-membered azonine ring of the mol­ecule adopts a *chair–boat* conformation (the basal planes are N4–C5/C1–C12*B* and C5–C6/C7*A*–C12*B*, respectively). It should be noted that the analogous nine-membered azonine ring in the related compound methyl 4-ethyl-11-methyl-1,4,5,6,7,8-hexa­hydro­azonino[5,6-*b*]indole-2-carboxyl­ate adopts a twisted *boat* conformation (Voskressensky, *et al.*, 2006 ▸). The C2=C3 and C3—N4 bond lengths [1.366 (3) and 1.407 (3) Å, respectively] indicate the presence of conjugation within the enamine C2=C3—N4 fragment. The substituent planes at the C2=C3 double bond are twisted by 16.0 (3)° because of steric effects. The N4 nitro­gen atom has a trigonal–pyramidal configuration (sum of the bond angles is 346.3°). The inter­planar angle between the two carboxyl­ate substituents is 60.39 (8)°.

## Supra­molecular features   

**Figure 3:**
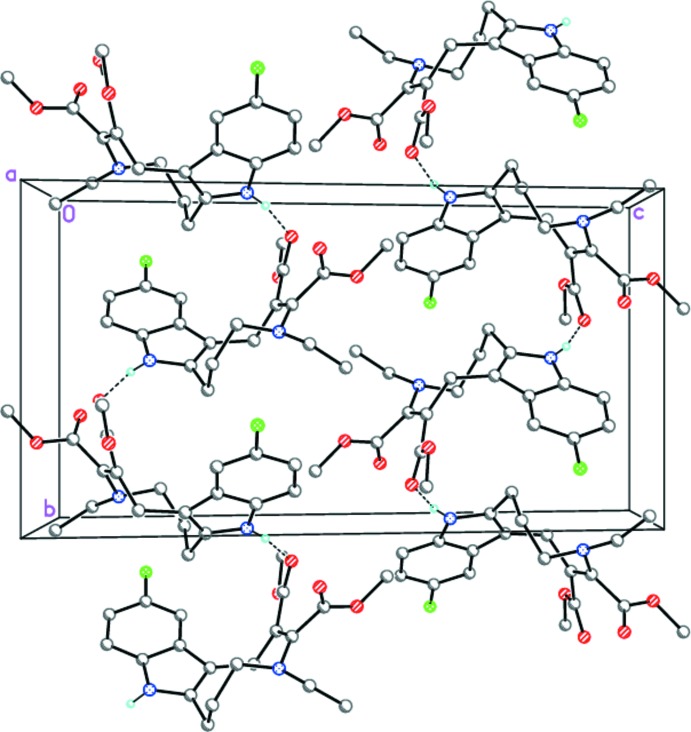
The crystal packing of **I** viewed along the *a*-axis direction showing the zigzag chains along [010]. Dashed lines indicate inter­molecular N—H⋯O hydrogen bonds.

In the crystal, mol­ecules of **I** form zigzag chains along [010] by inter­molecular N—H⋯O^i^ hydrogen-bonding inter­actions (Table 1 ▸, Fig. 3 ▸), which are further packed in stacks towards [100].

## Synthesis and crystallization   

Dimethyl acetyl­enedi­carboxyl­ate (170 mg, 1.2 mmol) was added to 2-ethyl-9-fluoro-1,2,3,4,5,6-hexa­hydro­azepino[4,3-*b*]indole (232 mg, 1 mmol) dissolved in methanol (10 ml). The reaction mixture was stirred for 2 h at room temperature with the TLC real-time control. Then the solvent was removed *in vacuo* and the residue was chromatographed over silica with ethyl­acetate:hexane as eluent to yield the target fluorinated azonino­indole **I** (22%) and 3-meth­oxy­methyl­indole **II**. Light-yellow crystals of azonino­indole **I** suitable for X-ray crystallographic analysis were grown by slow evaporation of an ethyl­acetate:hexane (1:1) solution, m.p. 456–458 K.

^1^H NMR (CDCl_3_, δ/ppm, *J*/Hz): 0.98 (*t*, 3H, *J* = 7.2, CH_3_CH_2_), 1.78 (*m*, 2H, 6-CH_2_), 2.74 (*q*, 2H, *J* = 7.2, CH_3_CH_2_), 2.93 (*m*, 2H, 7-CH_2_), 3.06 (*m*, 2H, 5-CH_2_), 3.96 (*s*, 2H, 1-CH_2_), 3.74 (*s*, 3H, CO_2_CH_3_), 3.77 (*s*, 3H, CO_2_CH_3_), 6.82 (*ddd*, 2H, ^1,3^ *J* = 9.0, ^1,3^ *J* = 9.0, ^1,4^ *J* = 2.3, CH-Ar), 7.13 (*m*, 2H, CH-Ar), 7.74 (*br s* 1H, NH). ^13^C NMR (DMSO-*d*_6_, δ/ppm, *J*/Hz): 15.2 (CH_3_), 21.9 (CH_2_), 23.8 (CH_2_), 27.1 (CH_2_), 44.5 (CH_2_), 52.3 (CH_3_), 52.3 (CH_3_), 55.5 (CH2), 102.5 (*d*, *J* = 22, CH), 108.2 (*d*, *J* = 26, CH), 108.6 (C), 111.8 (*d*, *J* = 9, CH), 122.3 (C), 128.3 (C), 132.2 (C), 137.9 (C), 151.7 (C), 157.1 (*d*, *J* = 231, C), 166.4 (C), 169.3 (C). IR (KBr): *ν* (cm^−1^) = 1723, 3373. Found (%): C, 64.16; H, 6.19; N, 7.48. C_20_H_23_FN_2_O_4_. Calculated (%): C, 64.46; H, 6.86; N, 7.82. Mass-spectrometry, *m*/*z* [I_rel_(%)]: 374 [M^+^] (100), 345 (20), 315 (100), 285 (30), 227 (10), 198 (20), 174 (30), 161 (30), 148 (10), 58 (40), 45 (10).

## Refinement   

Crystal data, data collection and structure refinement details are summarized in Table 2 ▸. The X-ray diffraction study was carried out on the "Belok" beamline of the National Research Center "Kurchatov Institute" (Moscow, Russian Federation) using a Rayonix SX165 CCD detector. A total of 360 images were collected using an oscillation range of 1.0° (φ scan mode, two different crystal orientations) and corrected for absorption using the *SCALA* program (Evans, 2006 ▸). The data were indexed, integrated and scaled using the utility *i*MOSFLM in the CCP4 program suite (Battye *et al.*, 2011 ▸).

The hydrogen atoms of the amino groups were localized in the difference-Fourier map and refined isotropically with fixed displacement parameters [*U*_iso_(H) = 1.2*U*_eq_(N)]. The other hydrogen atoms were placed in calculated positions with C—H = 0.95–0.99 Å and refined in the riding model with fixed isotropic displacement parameters [*U*_iso_(H) = 1.2*U*_eq_(C)].

## Supplementary Material

Crystal structure: contains datablock(s) global, I. DOI: 10.1107/S2056989018001329/kq2018sup1.cif

Structure factors: contains datablock(s) I. DOI: 10.1107/S2056989018001329/kq2018Isup2.hkl

Supporting information file. DOI: 10.1107/S2056989018001329/kq2018Isup3.cml

CCDC reference: 1818381

Additional supporting information:  crystallographic information; 3D view; checkCIF report

## Figures and Tables

**Table 1:** Hydrogen-bond geometry (Å, °)

*D*—H⋯*A*	*D*—H	H⋯*A*	*D*⋯*A*	*D*—H⋯*A*
N8—H8⋯O1^i^	0.93 (3)	2.17 (3)	3.025 (3)	153 (2)

Symmetry code: (i) 
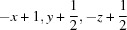
.

**Table 2:** Experimental details

Crystal data	
Chemical formula	C_20_H_23_FN_2_O_4_
*M*_r_	374.40
Crystal system, space group	Monoclinic, *P*2_1_/*c*
Temperature (K)	100
*a*, *b*, *c* (Å)	8.4632 (17), 10.993 (2), 20.520 (4)
β (°)	99.60 (3)
*V* (Å^3^)	1882.4 (7)
*Z*	4
Radiation type	Synchrotron, λ = 0.96990 Å
μ (mm^−1^)	0.21
Crystal size (mm)	0.22 × 0.02 × 0.02

Data collection	
Diffractometer	Rayonix SX165 CCD
Absorption correction	Multi-scan (*SCALA*; Evans, 2006 ▸)
*T*_min_, *T*_max_	0.940, 0.980
No. of measured, independent and observed [*I* > 2σ(*I*)] reflections	21117, 3850, 2463
*R*_int_	0.086
(sin θ/λ)_max_ (Å^−1^)	0.640

Refinement	
*R*[*F* ^2^ > 2σ(*F* ^2^)], *wR*(*F* ^2^), *S*	0.072, 0.184, 1.01
No. of reflections	3850
No. of parameters	251
H-atom treatment	H atoms treated by a mixture of independent and constrained refinement
Δρ_max_, Δρ_min_ (e Å^−3^)	0.34, −0.43

Computer programs: *MarCCD* (Doyle, 2011 ▸), *i* *MOSFLM* (Battye *et al.*, 2011 ▸), *SHELXT* (Sheldrick, 2015*a* ▸), *SHELXL2014* (Sheldrick, 2015*b* ▸) and *SHELXTL* (Sheldrick, 2008 ▸).

## supplementary crystallographic information

### Crystal data

**Table:**

C_20_H_23_FN_2_O_4_	*F*(000) = 792
*M**_r_* = 374.40	*D*_x_ = 1.321 Mg m^−^^3^
Monoclinic, *P*2_1_/*c*	Synchrotron radiation, λ = 0.96990 Å
*a* = 8.4632 (17) Å	Cell parameters from 600 reflections
*b* = 10.993 (2) Å	θ = 3.3–33.0°
*c* = 20.520 (4) Å	µ = 0.21 mm^−^^1^
β = 99.60 (3)°	*T* = 100 K
*V* = 1882.4 (7) Å^3^	Needle, yellow
*Z* = 4	0.22 × 0.02 × 0.02 mm

### Data collection

**Table:**

Rayonix SX165 CCD diffractometer	2463 reflections with *I* > 2σ(*I*)
/f scan	*R*_int_ = 0.086
Absorption correction: multi-scan (*SCALA*; Evans, 2006)	θ_max_ = 38.4°, θ_min_ = 3.3°
*T*_min_ = 0.940, *T*_max_ = 0.980	*h* = −10→10
21117 measured reflections	*k* = −12→10
3850 independent reflections	*l* = −26→25

### Refinement

**Table:**

Refinement on *F*^2^	Secondary atom site location: difference Fourier map
Least-squares matrix: full	Hydrogen site location: mixed
*R*[*F*^2^ > 2σ(*F*^2^)] = 0.072	H atoms treated by a mixture of independent and constrained refinement
*wR*(*F*^2^) = 0.184	*w* = 1/[σ^2^(*F*_o_^2^) + (0.090*P*)^2^] where *P* = (*F*_o_^2^ + 2*F*_c_^2^)/3
*S* = 1.01	(Δ/σ)_max_ < 0.001
3850 reflections	Δρ_max_ = 0.34 e Å^−^^3^
251 parameters	Δρ_min_ = −0.43 e Å^−^^3^
0 restraints	Extinction correction: SHELXL, Fc^*^=kFc[1+0.001xFc^2^λ^3^/sin(2θ)]^-1/4^
Primary atom site location: difference Fourier map	Extinction coefficient: 0.016 (2)

### Special details

**Table:**

Geometry. All esds (except the esd in the dihedral angle between two l.s. planes) are estimated using the full covariance matrix. The cell esds are taken into account individually in the estimation of esds in distances, angles and torsion angles; correlations between esds in cell parameters are only used when they are defined by crystal symmetry. An approximate (isotropic) treatment of cell esds is used for estimating esds involving l.s. planes.

### Fractional atomic coordinates and isotropic or equivalent isotropic displacement parameters (Å^2^)

**Table:**

	*x*	*y*	*z*	*U*_iso_*/*U*_eq_	
F1	−0.11330 (17)	0.18565 (15)	0.14176 (8)	0.0428 (5)	
O1	0.35878 (18)	0.12235 (17)	0.40043 (8)	0.0254 (5)	
O2	0.13856 (17)	0.24026 (16)	0.37589 (8)	0.0256 (5)	
O3	0.72767 (18)	0.17340 (16)	0.45020 (8)	0.0265 (5)	
O4	0.54751 (18)	0.24913 (16)	0.50997 (7)	0.0245 (5)	
C1	0.3100 (3)	0.4423 (2)	0.33722 (11)	0.0220 (6)	
H1A	0.1961	0.4424	0.3430	0.026*	
H1B	0.3592	0.5174	0.3581	0.026*	
C2	0.3917 (3)	0.3338 (2)	0.37585 (11)	0.0197 (6)	
C3	0.5500 (3)	0.3353 (2)	0.40360 (11)	0.0197 (6)	
N4	0.6592 (2)	0.41914 (19)	0.38442 (9)	0.0215 (5)	
C5	0.7014 (3)	0.3978 (2)	0.31780 (11)	0.0225 (6)	
H5A	0.6179	0.3459	0.2920	0.027*	
H5B	0.8041	0.3528	0.3229	0.027*	
C6	0.7174 (3)	0.5148 (2)	0.27897 (12)	0.0265 (6)	
H6A	0.7461	0.4933	0.2356	0.032*	
H6B	0.8060	0.5642	0.3032	0.032*	
C7	0.5632 (3)	0.5921 (3)	0.26769 (12)	0.0249 (6)	
H7A	0.5426	0.6237	0.3107	0.030*	
H7B	0.5783	0.6625	0.2393	0.030*	
C7A	0.4212 (3)	0.5196 (2)	0.23562 (11)	0.0214 (6)	
N8	0.3906 (2)	0.5038 (2)	0.16787 (9)	0.0229 (5)	
H8	0.437 (3)	0.547 (2)	0.1368 (12)	0.028*	
C8A	0.2622 (3)	0.4257 (2)	0.15110 (11)	0.0225 (6)	
C9	0.1871 (3)	0.3838 (2)	0.08913 (12)	0.0268 (6)	
H9	0.2220	0.4098	0.0497	0.032*	
C10	0.0602 (3)	0.3033 (3)	0.08712 (13)	0.0299 (7)	
H10	0.0070	0.2722	0.0461	0.036*	
C11	0.0114 (3)	0.2683 (3)	0.14629 (13)	0.0283 (6)	
C12	0.0805 (3)	0.3085 (2)	0.20795 (12)	0.0248 (6)	
H12	0.0424	0.2828	0.2467	0.030*	
C12A	0.2112 (2)	0.3900 (2)	0.21103 (11)	0.0214 (6)	
C12B	0.3143 (3)	0.4501 (2)	0.26446 (11)	0.0201 (6)	
C13	0.2988 (3)	0.2225 (3)	0.38597 (11)	0.0221 (6)	
C14	0.0434 (3)	0.1308 (3)	0.38115 (13)	0.0292 (7)	
H14A	0.0642	0.0713	0.3480	0.044*	
H14B	−0.0707	0.1519	0.3735	0.044*	
H14C	0.0730	0.0958	0.4254	0.044*	
C15	0.6182 (3)	0.2417 (2)	0.45594 (11)	0.0212 (6)	
C16	0.5946 (3)	0.1559 (3)	0.55942 (12)	0.0295 (7)	
H16A	0.5529	0.0769	0.5422	0.044*	
H16B	0.5509	0.1755	0.5995	0.044*	
H16C	0.7118	0.1522	0.5700	0.044*	
C17	0.7970 (3)	0.4562 (3)	0.43429 (11)	0.0248 (6)	
H17A	0.8687	0.5087	0.4131	0.030*	
H17B	0.8583	0.3829	0.4514	0.030*	
C18	0.7454 (3)	0.5244 (3)	0.49167 (13)	0.0317 (7)	
H18A	0.6843	0.5969	0.4750	0.048*	
H18B	0.8404	0.5489	0.5230	0.048*	
H18C	0.6782	0.4714	0.5140	0.048*	

### Atomic displacement parameters (Å^2^)

**Table:**

	*U*^11^	*U*^22^	*U*^33^	*U*^12^	*U*^13^	*U*^23^
F1	0.0287 (8)	0.0431 (12)	0.0508 (10)	−0.0170 (7)	−0.0099 (7)	0.0069 (8)
O1	0.0152 (8)	0.0275 (12)	0.0328 (10)	0.0029 (8)	0.0016 (7)	0.0025 (8)
O2	0.0108 (8)	0.0301 (12)	0.0356 (10)	−0.0008 (7)	0.0034 (7)	0.0043 (8)
O3	0.0172 (8)	0.0318 (12)	0.0300 (10)	0.0066 (7)	0.0024 (7)	0.0008 (8)
O4	0.0215 (9)	0.0338 (12)	0.0189 (9)	0.0030 (7)	0.0052 (6)	0.0043 (8)
C1	0.0127 (11)	0.0284 (17)	0.0241 (13)	0.0018 (10)	0.0003 (9)	0.0018 (11)
C2	0.0133 (11)	0.0260 (16)	0.0195 (12)	−0.0001 (10)	0.0016 (9)	0.0000 (10)
C3	0.0147 (11)	0.0262 (16)	0.0181 (11)	0.0016 (10)	0.0020 (8)	−0.0007 (10)
N4	0.0135 (9)	0.0308 (14)	0.0194 (10)	−0.0041 (9)	0.0006 (7)	0.0001 (9)
C5	0.0147 (11)	0.0326 (17)	0.0198 (12)	0.0000 (10)	0.0018 (9)	−0.0001 (11)
C6	0.0165 (12)	0.0375 (18)	0.0243 (13)	−0.0068 (11)	0.0005 (9)	0.0023 (12)
C7	0.0205 (12)	0.0309 (17)	0.0222 (13)	−0.0044 (11)	0.0004 (10)	0.0032 (11)
C7A	0.0182 (12)	0.0251 (16)	0.0196 (13)	0.0021 (10)	−0.0007 (9)	0.0005 (11)
N8	0.0173 (10)	0.0313 (15)	0.0190 (11)	−0.0035 (9)	0.0000 (8)	0.0028 (10)
C8A	0.0137 (11)	0.0286 (17)	0.0235 (13)	0.0018 (10)	−0.0016 (9)	0.0001 (11)
C9	0.0201 (12)	0.0321 (19)	0.0267 (14)	0.0026 (11)	0.0000 (10)	0.0002 (12)
C10	0.0194 (12)	0.0363 (19)	0.0295 (14)	0.0012 (12)	−0.0088 (10)	−0.0024 (12)
C11	0.0147 (12)	0.0284 (18)	0.0377 (15)	−0.0034 (11)	−0.0073 (10)	0.0043 (13)
C12	0.0150 (11)	0.0277 (17)	0.0299 (14)	0.0019 (10)	−0.0015 (9)	0.0073 (12)
C12A	0.0122 (11)	0.0267 (17)	0.0237 (13)	0.0035 (10)	−0.0019 (9)	0.0048 (11)
C12B	0.0143 (11)	0.0261 (16)	0.0193 (12)	0.0040 (10)	0.0009 (9)	0.0045 (11)
C13	0.0116 (11)	0.0356 (19)	0.0182 (12)	0.0015 (11)	0.0000 (8)	−0.0008 (11)
C14	0.0144 (11)	0.0300 (18)	0.0441 (16)	−0.0057 (11)	0.0076 (10)	0.0043 (13)
C15	0.0141 (11)	0.0280 (17)	0.0204 (12)	−0.0026 (10)	−0.0001 (9)	−0.0015 (11)
C16	0.0231 (13)	0.0402 (19)	0.0229 (13)	−0.0009 (12)	−0.0027 (10)	0.0095 (12)
C17	0.0159 (11)	0.0321 (17)	0.0248 (13)	−0.0033 (11)	−0.0013 (9)	0.0019 (11)
C18	0.0208 (12)	0.0424 (19)	0.0305 (14)	−0.0068 (12)	−0.0002 (10)	−0.0059 (13)

### Geometric parameters (Å, º)

**Table:**

F1—C11	1.383 (3)	C7A—N8	1.382 (3)
O1—C13	1.228 (3)	C7A—C12B	1.390 (3)
O2—C13	1.352 (3)	N8—C8A	1.383 (3)
O2—C14	1.461 (3)	N8—H8	0.93 (3)
O3—C15	1.213 (3)	C8A—C9	1.401 (3)
O4—C15	1.347 (3)	C8A—C12A	1.425 (3)
O4—C16	1.451 (3)	C9—C10	1.388 (3)
C1—C12B	1.502 (3)	C9—H9	0.9500
C1—C2	1.532 (3)	C10—C11	1.400 (4)
C1—H1A	0.9900	C10—H10	0.9500
C1—H1B	0.9900	C11—C12	1.376 (3)
C2—C3	1.366 (3)	C12—C12A	1.417 (3)
C2—C13	1.487 (4)	C12—H12	0.9500
C3—N4	1.407 (3)	C12A—C12B	1.442 (3)
C3—C15	1.530 (3)	C14—H14A	0.9800
N4—C17	1.475 (3)	C14—H14B	0.9800
N4—C5	1.488 (3)	C14—H14C	0.9800
C5—C6	1.531 (4)	C16—H16A	0.9800
C5—H5A	0.9900	C16—H16B	0.9800
C5—H5B	0.9900	C16—H16C	0.9800
C6—C7	1.542 (3)	C17—C18	1.520 (4)
C6—H6A	0.9900	C17—H17A	0.9900
C6—H6B	0.9900	C17—H17B	0.9900
C7—C7A	1.499 (3)	C18—H18A	0.9800
C7—H7A	0.9900	C18—H18B	0.9800
C7—H7B	0.9900	C18—H18C	0.9800
			
C13—O2—C14	114.88 (19)	C8A—C9—H9	121.0
C15—O4—C16	115.13 (19)	C9—C10—C11	119.3 (2)
C12B—C1—C2	118.4 (2)	C9—C10—H10	120.4
C12B—C1—H1A	107.7	C11—C10—H10	120.4
C2—C1—H1A	107.7	C12—C11—F1	118.4 (2)
C12B—C1—H1B	107.7	C12—C11—C10	124.6 (2)
C2—C1—H1B	107.7	F1—C11—C10	117.0 (2)
H1A—C1—H1B	107.1	C11—C12—C12A	117.0 (2)
C3—C2—C13	117.2 (2)	C11—C12—H12	121.5
C3—C2—C1	122.2 (2)	C12A—C12—H12	121.5
C13—C2—C1	120.61 (19)	C12—C12A—C8A	118.8 (2)
C2—C3—N4	122.4 (2)	C12—C12A—C12B	133.8 (2)
C2—C3—C15	120.9 (2)	C8A—C12A—C12B	107.3 (2)
N4—C3—C15	116.65 (18)	C7A—C12B—C12A	106.40 (19)
C3—N4—C17	117.83 (18)	C7A—C12B—C1	125.5 (2)
C3—N4—C5	115.06 (19)	C12A—C12B—C1	128.1 (2)
C17—N4—C5	113.43 (16)	O1—C13—O2	122.0 (2)
N4—C5—C6	113.7 (2)	O1—C13—C2	124.4 (2)
N4—C5—H5A	108.8	O2—C13—C2	113.6 (2)
C6—C5—H5A	108.8	O2—C14—H14A	109.5
N4—C5—H5B	108.8	O2—C14—H14B	109.5
C6—C5—H5B	108.8	H14A—C14—H14B	109.5
H5A—C5—H5B	107.7	O2—C14—H14C	109.5
C5—C6—C7	113.24 (19)	H14A—C14—H14C	109.5
C5—C6—H6A	108.9	H14B—C14—H14C	109.5
C7—C6—H6A	108.9	O3—C15—O4	124.7 (2)
C5—C6—H6B	108.9	O3—C15—C3	123.6 (2)
C7—C6—H6B	108.9	O4—C15—C3	111.68 (19)
H6A—C6—H6B	107.7	O4—C16—H16A	109.5
C7A—C7—C6	111.7 (2)	O4—C16—H16B	109.5
C7A—C7—H7A	109.3	H16A—C16—H16B	109.5
C6—C7—H7A	109.3	O4—C16—H16C	109.5
C7A—C7—H7B	109.3	H16A—C16—H16C	109.5
C6—C7—H7B	109.3	H16B—C16—H16C	109.5
H7A—C7—H7B	107.9	N4—C17—C18	112.19 (18)
N8—C7A—C12B	109.5 (2)	N4—C17—H17A	109.2
N8—C7A—C7	120.7 (2)	C18—C17—H17A	109.2
C12B—C7A—C7	129.5 (2)	N4—C17—H17B	109.2
C7A—N8—C8A	109.62 (19)	C18—C17—H17B	109.2
C7A—N8—H8	126.5 (15)	H17A—C17—H17B	107.9
C8A—N8—H8	123.3 (15)	C17—C18—H18A	109.5
N8—C8A—C9	130.4 (2)	C17—C18—H18B	109.5
N8—C8A—C12A	107.2 (2)	H18A—C18—H18B	109.5
C9—C8A—C12A	122.4 (2)	C17—C18—H18C	109.5
C10—C9—C8A	117.9 (2)	H18A—C18—H18C	109.5
C10—C9—H9	121.0	H18B—C18—H18C	109.5
			
C12B—C1—C2—C3	88.1 (3)	N8—C8A—C12A—C12	−179.6 (2)
C12B—C1—C2—C13	−93.4 (3)	C9—C8A—C12A—C12	0.4 (4)
C13—C2—C3—N4	163.5 (2)	N8—C8A—C12A—C12B	0.1 (3)
C1—C2—C3—N4	−18.0 (3)	C9—C8A—C12A—C12B	−179.9 (2)
C13—C2—C3—C15	−14.3 (3)	N8—C7A—C12B—C12A	−0.9 (3)
C1—C2—C3—C15	164.2 (2)	C7—C7A—C12B—C12A	−174.2 (2)
C2—C3—N4—C17	150.5 (2)	N8—C7A—C12B—C1	179.6 (2)
C15—C3—N4—C17	−31.6 (3)	C7—C7A—C12B—C1	6.3 (4)
C2—C3—N4—C5	−71.5 (3)	C12—C12A—C12B—C7A	−179.9 (2)
C15—C3—N4—C5	106.4 (2)	C8A—C12A—C12B—C7A	0.5 (3)
C3—N4—C5—C6	141.0 (2)	C12—C12A—C12B—C1	−0.4 (4)
C17—N4—C5—C6	−79.1 (2)	C8A—C12A—C12B—C1	−180.0 (2)
N4—C5—C6—C7	−58.9 (2)	C2—C1—C12B—C7A	−98.2 (3)
C5—C6—C7—C7A	−54.3 (3)	C2—C1—C12B—C12A	82.3 (3)
C6—C7—C7A—N8	−83.2 (3)	C14—O2—C13—O1	−2.1 (3)
C6—C7—C7A—C12B	89.4 (3)	C14—O2—C13—C2	176.11 (18)
C12B—C7A—N8—C8A	1.0 (3)	C3—C2—C13—O1	−21.2 (3)
C7—C7A—N8—C8A	174.9 (2)	C1—C2—C13—O1	160.2 (2)
C7A—N8—C8A—C9	179.3 (3)	C3—C2—C13—O2	160.6 (2)
C7A—N8—C8A—C12A	−0.6 (3)	C1—C2—C13—O2	−18.0 (3)
N8—C8A—C9—C10	179.1 (2)	C16—O4—C15—O3	−8.8 (3)
C12A—C8A—C9—C10	−1.0 (4)	C16—O4—C15—C3	174.42 (19)
C8A—C9—C10—C11	0.7 (4)	C2—C3—C15—O3	122.1 (3)
C9—C10—C11—C12	0.2 (4)	N4—C3—C15—O3	−55.9 (3)
C9—C10—C11—F1	−178.6 (2)	C2—C3—C15—O4	−61.1 (3)
F1—C11—C12—C12A	178.0 (2)	N4—C3—C15—O4	121.0 (2)
C10—C11—C12—C12A	−0.7 (4)	C3—N4—C17—C18	−64.2 (3)
C11—C12—C12A—C8A	0.4 (3)	C5—N4—C17—C18	157.2 (2)
C11—C12—C12A—C12B	−179.2 (3)		

### Hydrogen-bond geometry (Å, º)

**Table:**

*D*—H···*A*	*D*—H	H···*A*	*D*···*A*	*D*—H···*A*
N8—H8···O1^i^	0.93 (3)	2.17 (3)	3.025 (3)	153 (2)

Symmetry code: (i) −*x*+1, *y*+1/2, −*z*+1/2.
